# The impact of mechanical tuning on the printability of decellularized amniotic membrane bioinks for cell-laden bioprinting of soft tissue constructs

**DOI:** 10.1038/s41598-024-80973-3

**Published:** 2024-11-29

**Authors:** Golara Kafili, Elnaz Tamjid, Abdolreza Simchi

**Affiliations:** 1 Center for Nanoscience and Nanotechnology, Institute for Convergence Science & Technology, Sharif University of Technology, P.O. Box 14588-89694, Tehran, Iran; 2 Department of Nanobiotechnology, Faculty of Biological Sciences, Tarbiat Modares University, P.O. Box 14115-175, Tehran, Iran; 3 Advanced Ceramics, University of Bremen, 28359 Bremen, Germany; 4 Department of Materials Science and Engineering, Sharif University of Technology, Azadi Avenue, P.O. Box 11365-11155, Tehran, Iran; 5 Center for BioScience and Technology, Institute for Convergence Science & Technology, Sharif University of Technology, P.O. Box 14588-89694, Tehran, Iran; 6 Present Address: Fraunhofer Institute for Manufacturing Technology and Advanced Materials (IFAM), 28359 Bremen, Germany

**Keywords:** Decellularized extracellular matrix, Bioink, Printability, Rheology, Proliferation, Regenerative medicine, Tissues, Biomaterials - cells

## Abstract

Decellularized extracellular matrix (dECM) bioinks hold significant potential in the 3D bioprinting of tissue-engineered constructs (TECs). While 3D bioprinting allows for the creation of custom-designed TECs, the development of bioinks based solely on dAM, without the inclusion of supporting agents or chemical modifications, remains underexplored. In this study, we present the concentration-dependent printability and rheological properties of dAM bioinks, along with an analysis of their in vitro cellular responses. Our findings demonstrate that increasing dAM concentrations, within the range of 1 to 3% w/v, enhances the mechanical moduli of the bioinks, enabling the 3D printing of flat structures with superior shape fidelity. In vitro assays reveal high cell viability across all dAM bioink formulations; however, at 3% w/v, the bioink tends to impede fibroblast proliferation, resulting in round cell morphology. We propose that bioinks containing 2% w/v dAM strike an optimal balance, providing fine-resolved features and a supportive microenvironment for fibroblasts, promoting elongated spindle-like morphology and enhanced proliferation. These results underscore the importance of dAM concentration in regulating the properties and performance of bioinks, particularly regarding cell viability and morphology, for the successful 3D bioprinting of soft tissues.

## Introduction

3D bioprinting is a well-developed method for engineering tissues by recapitulating anatomical architecture or preparing in vitro platforms for studying cell-related biological phenomena^1^. Among various types of biomaterials exploited as bioinks, the decellularized extracellular matrix (dECM)-derived hydrogels are a promising source of bioinks with tissue-specific biochemical cues that have been widely used in tissue engineering (TE) in the recent decade^2^. Bioprinting of dECM bioinks is performed to mimic biophysical cues of target tissues by constructing anatomically relevant geometries^3^. For example, bioprinting of stem cell-embedded methacrylated muscle dECM bioink (MdECMMA) coupled with in situ electrical stimulation has been examined for muscle tissue engineering^4^. The bioprinted muscle-like constructs guide the formation of aligned cytoskeletons and support the expression of myogenic genes. The potential of 3D bioprinting for fabricating in vitro models of healthy or diseased tissues has also been demonstrated. Shin and colleagues^5^have reported that heart dECM bioink can be tailored by adding polyethylene glycol diacrylate (PEGDA) and laponite nanoplatelets. The resulting nanocomposite bioinks can be used to 3D print constructs with mechanical moduli ranging from 5–15 kPa and 30–100 kPa, mimicking the properties of healthy and fibrotic cardiac tissues, respectively. However, so far only a few studies have investigated the effect of digesting conditions of decellularized tissues and organs on the printability of dECM-derived bioinks^6–9^. For instance, Zhao et al.^6^ have shown that the relatively short time digestion (three hours) of tendon provides superior printability and shape fidelity compared to prolonged digestion times (72 h), owing to its higher viscoelasticity.

Among various types of tissues and organs, the amniotic membrane (AM) is a desirable tissue source owing to its availability, abundance, cost-effectiveness, and exquisite biological properties, including anti-fibrotic, angiogenic, antimicrobial, anti-inflammatory, and low immunogenicity^10^. However, the difficulty in handling fresh AM and suturing it on irregularly shaped tissue defects or cavities restricts its applicability^11^. In recent years, processing the decellularized amniotic membrane (dAM) into a thermosensitive and injectable hydrogel has been pursued to solve this issue^12,13^. The dAM hydrogels have shown promising therapeutic outcomes in several TE fields, such as skin regeneration^14^, heart repair^15^, vascular graft^16^, cartilage regeneration^17^, and cell delivery^18^, owing to the preservation of bioactive components within the dAM matrix during processing steps.

Despite the recent interest in developing dAM hydrogels for various TE applications, their potential for 3D bioprinting has not yet been thoroughly studied and explored. Heidari et al.^19^ have utilized coaxial extrusion-based bioprinting for the fabrication of vascularized tissue-engineered constructs (TECs). They have used cell-encapsulated sodium alginate containing dAM particulates as sheath bioink and CaCl_2_crosslinking solution as core material. The results of the study showed that the 3D-printed constructs support the activity of endothelial cells, including cell viability, proliferation, migration, and tubulogenesis, confirming their potential for angiogenesis. Nevertheless, the incorporation of dAM particulates in a hydrogel instead of solubilizing it to form a flowing hydrogel as bioink can cause heterogeneity in bioink formulation and more importantly, lead to severe nozzle clogging during the printing process^19^. In another study, bioinks derived from methacrylated amniotic membrane (AdECMMA) and methacrylated chorionic membrane (CdECMMA) reinforced with methacrylated hyaluronic acid (HAMA) were compared for their printability and potential angiogenic properties^20^. The hybrid AdECMMA-HAMA and CdECMMA-HAMA bioinks did not exhibit significant differences in terms of rheological characteristics and printability, while both materials supported in vitro vasculogenesis. Despite the promising results obtained for AdECMMA-HAMA and CdECMMA-HAMA bioinks, the methacrylation of dECM materials could harm the structural integrity of dECM-derived hydrogels and diminish their bioactivity^21^. As a result, the AdECMMA or CdECMMA hydrogels did not provide enough viscosity and rheological properties for the 3D bioprinting process and inevitably were mixed with HAMA to replenish their lack of printability^20^. In another study, a 3D-printed polycaprolactone (PCL) framework encompassing dAM hydrogel was designed as an amnion-analogous medical device (AMED) to repair amniotic membrane ruptures and sealing of the amniotic fluid^22^. The insertion of the medical device via fetoscopic procedure was examined on pregnant swine models. Although the 3D-printed AMED provided proper handling and therapeutic outcomes, the PCL supportive structure was inevitable to render dAM hydrogel with adequate stability and handling.

Recently, a printable dAM-based nanoengineered ink formulation for wound healing applications has been developed^23^. In the formulation, sodium alginate is incorporated as a structurally supportive component to endow descent printability. Laponite nanoplatelets are also added as a rheology modifying agent to enhance the shape fidelity and self-standing characteristic of the bioink for printing tubular constructs without compromising biological properties. In another study, hydroxyethyl cellulose (HEC) was added as a thickening agent to the dAM bioink to improve the poor extrudability and printability of the pure dAM bioink^24^. Although promising results have been achieved for the in vitro wound healing process, the encapsulation of cells into the ink formulations has not been examined. Cell encapsulation is important because it enables the deposition of cells in the desired pre-defined location and facilitates a more precise emulation of the architectural features of native tissues^25^.

**Fig. 1:**
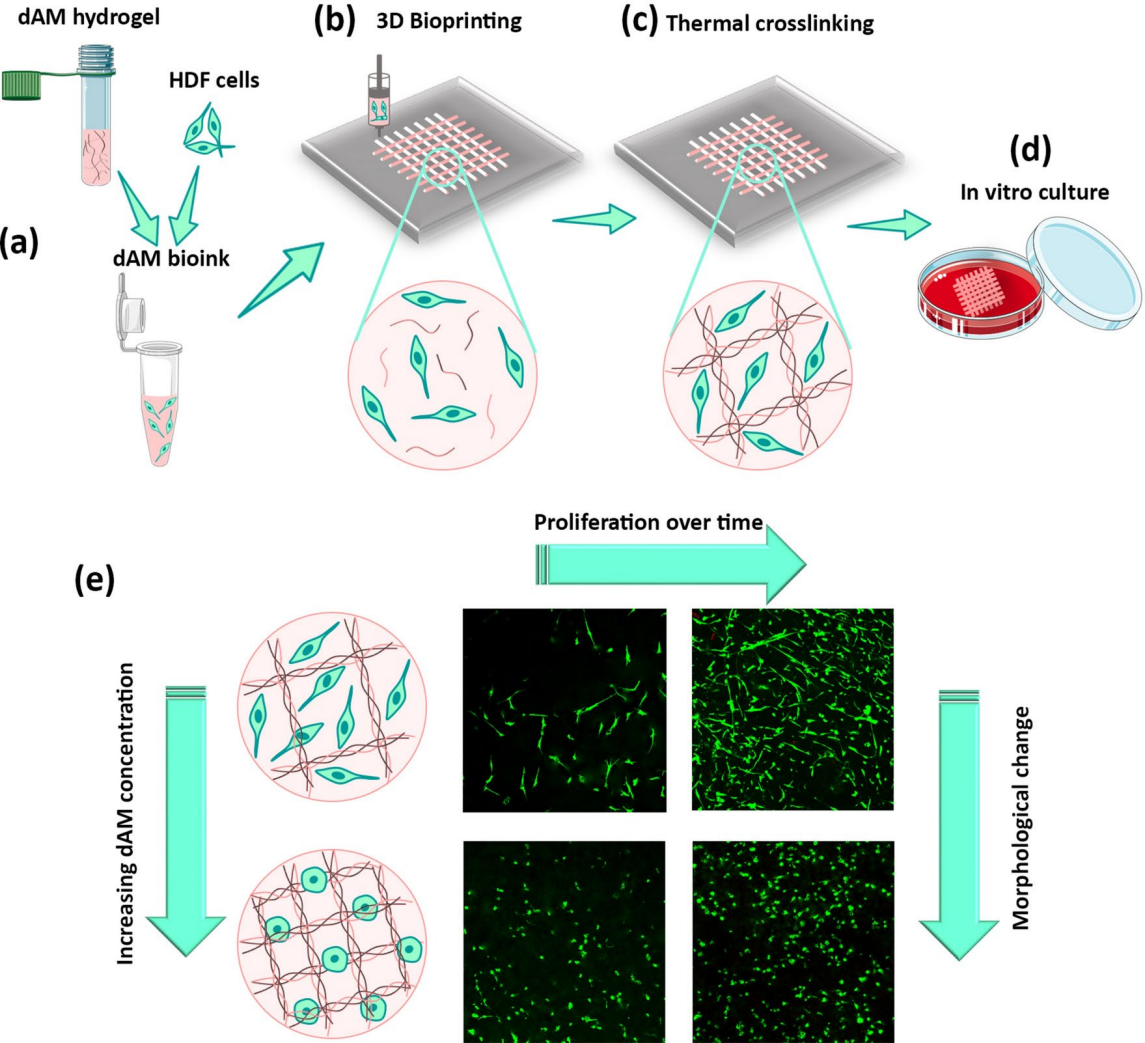
Preparation of cell-laden constructs using decellularized amniotic membrane (dAM) bioink. (**a**) Processing cell-laden bioinks by mixing human dermal fibroblast (HDF) cells with neutralized dAM hydrogel to prepare a printable bioink. (**b**) 3D bioprinting of the desired pattern using dAM bioink. (**c**) Self-assembly of collagen fibers to provide thermosensitive response to dAM bioinks for thermal crosslinking (at 37˚C) of 3D bioprinted constructs. (**d**) In vitro culture of bioprinted tissue-engineered constructs (TECs) in culture medium. (**e**) Morphological changes of HDF cells encapsulated in dAM bioinks in response to dAM concentration. Schemes were generated using Servier Medical Art licensed under a Creative Commons Attribution 4.0.

It is important to warrant the reliability and reproducibility of the biofabrication process to ensure the functionality and fidelity of scaffolds for TE applications. Considering the fascinating potential of dECM-derived bioinks for replicating the tissue-specific features of the target tissues, there has been a growing trend of reports about the printability of dECM biomaterials derived from various tissue sources^26^. However, to the best of our knowledge, the printability of pure dAM bioink without the inclusion of supporting polymer components or any further chemical modifications has not been reported yet. This study aims to investigate the printability of dAM bioinks containing different dAM concentrations without the administration of supportive or rheology-modifying components. Additionally, fibroblast cells are encapsulated in the bioink and their survival and proliferative capacity are thoroughly assessed (Fig. 1a). The thermoresponsive characteristic of solubilized dAM caused by the self-assembly of collagen fibers enables the thermal crosslinking of bioprinted constructs by incubation at 37°C (Figs. 1b and c**).** The main novelty of this work relies on the 3D bioprinting of cell-laden dAM bioinks without additional interventions, such as the methacrylation process to endow photo-crosslinkability and/or usage of an auxiliary polymeric component. Our understanding shed light on a roadmap to develop thermosensitive dAM bioinks with good shape fidelity and cell viability. It is shown that the mechanical properties of dAM bioinks can be tuned by altering the dAM concentration, which ultimately affects the fibroblast cell response, including viability, proliferation, and morphology (Fig. 1e). Tailoring the mechanical, rheological, and biological properties of pristine dAM bioinks by changing synthesis parameters, such as concentration, instead of the inclusion of any further aiding agents (e.g. structural polymers, nanomaterials, or photoactive components) would help to widen its therapeutic and biomedical applications without concerning any undesirable side effects raised by complementary additives. Therefore, this work aims to evaluate the effectiveness of the extrusion-based 3D bioprinting technique on the shape fidelity and architectural integrity of tissue-engineered constructs (TECs) prepared using dAM bioinks as a versatile, available, and bioactive source. In this context, we have examined the effect of dAM concentration, printing pressure, and designed feature size on the printing fidelity of the TECs to capture a universal application window of dAM bioinks for potential use in the TE field. The results would have important implications for the 3D bioprinting of dAM bioinks and regulating fibroblast cells for soft tissue engineering applications.

## Materials and methods

### Materials

Dulbecco’s phosphate-buffered saline (DPBS) liquid, Dulbecco’s Modified Eagle’s medium (DMEM)/high glucose, Penicillin/Streptomycin antibiotic, and Fetal bovine serum (FBS) were obtained from HyClone (Cytiva, USA). Pepsin enzyme (P7125), proteinase K, and Peracetic acid (PAA) were provided by Sigma-Aldrich (St. Louis, MO, USA). Trypsin/EDTA was purchased from Gibco (USA). Ethylenediaminetetraacetic acid (EDTA) was purchased from Tech & Innovation company (T&I, Chuncheon Bioindustry Foundation, Gangwon, South Korea). Triton X-100 and 10N NaOH solution were obtained from Biosesang company (Sungnam, Korea). Type I atelocollagen from porcine skin was purchased from Dalim Tissen Co. (Seoul, Korea). Cell counting kit-8 (CCK-8) was obtained from Dojindo laboratories (Japan).

### Preparation of dAM-derived hydrogels

A schematic representation of preparing dAM bioink for 3D bioprinting of custom-designed TECs for soft tissue engineering is shown in Fig. 1. The porcine placentas were obtained from local farms and carried on ice to the laboratory. The decellularization process was carried out according to the protocol published by Lee et al.^22^ with minor modifications. Briefly, the AM tissue was separated from the chorionic membrane and rinsed several times in a sterile PBS solution to wash out the blood clots. Next, the AM was cut into approximately 4 cm^2^ pieces and treated with 0.2%w/v EDTA solution for 2 h at 37 °C. After washing with PBS to remove cell fragments, the tissue pieces were incubated in 1%v/v Triton X-100 for 4 h at room temperature (RT). The decellularized tissues were washed three times with PBS and sterilized with PAA (0.1%w/v) dissolved in an ethanol solution (4%) for 2 h. Lyophilized dAM tissue pieces were then grounded using a grinding device (POWTEQ, FM200 model, China) for 3 min.

The dAM solutions were prepared by digestion of the required amount of dAM powder in HCl solution with a concentration of 0.01 M and supplemented with 10%w/w pepsin enzyme for 5 days at room temperature. Pre-gel solutions with a final concentration of 1, 2, and 3%w/v were neutralized using 10N NaOH and salt balanced. The obtained hydrogels are accordingly named as AM1, AM2, and AM3. The neutralized pre-gel solutions were capable of gelation by thermally crosslinking at 37 ˚C for 1 h.

### Biochemical characterization of dAM tissue

DNA, GAG, and collagen content of dAM tissue were quantified and compared to native AM tissue to assess the quality of the decellularization process. For quantifying the double-stranded DNA (ds-DNA) content, DNA from tissues was extracted using a GeneJET genomic DNA purification kit (ThermoFisher Scientific, USA) and quantified using Quant-iT PicoGreen ds-DNA assay kit (Invitrogen Life Technologies, USA) based on the manufacturer’s protocol. The fluorescence emissions were recorded using a Synergy H1 fluorescence microplate reader (BioTek, USA). For GAG and collagen quantification, the tissues were solubilized in a papain solution (125 mg/mL papain in 0.1 M sodium phosphate with 5 mM Na_2_-EDTA and 5 mM cysteine-HCl (pH = 6.5)) at 60 °C. The sGAG and collagen contents were determined using the 1,9-dimethyl methylene blue solution and colorimetric hydroxyproline assay kit (Biovision, USA) according to the manufacturer’s instructions. The absorbance of samples was measured using a microplate reader (ThermoFisher Scientific, Multiskan GO, USA) at a wavelength of 525 nm and 560 nm for the quantification of GAG and collagen, respectively. In all cases, the obtained values were normalized to the value of native tissue, and the biochemical results were reported as percentages.

### Rheological studies

The rheological properties of dAM hydrogels were performed using a TA instrument rheometer (USA) with a 25 mm cone plate geometry and 500 µm gap. The frequency sweep test was conducted in the range of 10^–2^−10^2^ rad/s at a constant strain of 1% after incubation of pre-gel solutions at 37 ˚C for 30 min. A steady shear sweep analysis in the range of 10^–1^−10^3^ s^−1^at 4 °C was applied to obtain the viscosity of pre-gel solutions. The shear stress (τ)-shear rate (γ) curves were fitted with the following rheological models^27^:

1 $${\text{Bingham plastic model}}: \, \tau \, = \, \tau_{{\text{y}}} + \, \kappa \gamma$$

2 $${\text{Herschel}} - {\text{Bulkley model}}: \, \tau \, = \, \tau_{{\text{y}}} + \, \kappa \, \left( \gamma \right)^{{\text{n}}}$$

3 $${\text{Casson model}}: \, \tau^{{{1}/{2}}} = \, \tau_{{\text{y}}}^{{{1}/{2}}} + \, \kappa \, \left( \gamma \right)^{{{1}/{2}}}$$

*k*, *n*, and *τ*_*y*_ are the flow consistency index, shear thinning index, and yield stress, respectively.

### 3D printing

A 3D bioprinter (T&R company, Korea) equipped with a pneumatic pressure controller (Musashi Engineering Inc., Japan) was used for printing dAM hydrogels. The hydrogels were loaded into a syringe with a 24G needle (inner diameter of 0.31 mm) and installed in a printer dispenser. The temperature of the print head was set to 4°C in order to inhibit gelation before the printing process. The temperature of the printing bed was set to 37°C to allow the gelation of dAM hydrogels after deposition. The printability was assessed at different applied pressures using a lattice structure. The printability parameter (Pr) and circularity (C) of the pore area inside the lattice design were calculated using the following equations^28^:

4 $$\text{C}=\frac{4\uppi \times \text{pore area}}{{\text{pore perimeter}}^{2}}$$

5 $$\text{Pr }=\frac{{\text{pore perimeter}}^{2}}{16 \times \text{pore area}}$$

For the assessment of shape fidelity, different shapes, including squares, stars, and circles were printed. A digital microscope (Dino-Lite) was employed to image the printed structures. The ratio of the actual area to the theoretical area (RAATA) was calculated by an image analyzer (Digimizer, version 5.4.9). For the stacking test, ten alternative layers of hollow tubular structures with a diameter of 10 mm and a designed height of 0.31 mm for each layer were printed and the height of the printed tubes was determined using the Digimizer image analyzer.

### In vitro* assays*

#### Cell culture

Human dermal fibroblast cells (HDFs, Lonza, Switzerland) were cultured with DMEM, 10% FBS, and 1% penicillin–streptomycin antibiotic. The cells were cultured until reaching about 80–90% confluency, while the medium was refreshed every other day. Afterward, the cells were dissociated from the culture flask using 0.25% trypsin/EDTA and centrifuged at 1500 rpm for 5 min. The collected HDF cells with the desired cell density were resuspended in a complete culture medium for further use.

#### Live/dead cell viability

HDFs with a cell density of 10^6^ cells/mL were encapsulated in dAM hydrogels with a final concentration of 1, 2, and 3%w/v. The HDF-encapsulated collagen hydrogel at a final concentration of 2% w/v served as the 3D control group, while HDF cells seeded on tissue culture plates were used as the 2D control group. Cell-laden hydrogels (100 µL/well) were gently pipetted into a 24-well plate and incubated at 37 °C for 60 min. Subsequently, 1 mL complete medium was added to each well and incubated for 1, 3, and 7 days. The medium was refreshed every other day. After specific time intervals, the medium was removed, and cell-encapsulated hydrogels along with control groups were rinsed with PBS and stained with Calcein-AM and ethidium homodimer solutions (Live/Dead assay kit, Thermo Fisher Scientific, USA) for 30 min. Live and dead cells were visualized in green and red colors using a confocal microscope (Nikon Instruments, C2 model, Japan). The size of the cell-encapsulated hydrogels with an initial volume of 100 µL/well was monitored by a Digimizer image analyzer to determine the cell-mediated contractile behavior over time.

#### Cell proliferation

To assess the proliferation activity of cells, HDFs with a cell density of 10^6^ cells/mL were encapsulated in dAM hydrogels with a final concentration of 1, 2, and 3%w/v. The HDF cells, at the same density, were encapsulated in a 2% w/v collagen hydrogel and seeded onto tissue culture plates, serving as the 3D and 2D control groups, respectively. Then, 100 µl/well cell-encapsulated pre-gel solutions were coated in 48-well plates and incubated for one hour at 37 °C. After gelation, 500 µl medium was added to each well and incubated for 7 days while exchanging the medium. Three replicates were considered for each condition. The medium was discarded at a given time interval and the samples were treated with 10% CCK-8 solution for 4 h at 37°C while protected from light. The absorbance of the solutions was recorded at the wavelength of 450 nm using a microplate reader (Thermo Scientific, Multiscan GO model, USA).

### 3D bioprinting

Lattice structures with different designed gap sizes between lines (i.e. feature size) were printed by AM2 bioink with a cell density of 1 × 10^6^ HDFs/mL using pneumatic pressure of 10 and 20 kPa. The constructs with a feature size of 2 mm were printed using the AM2 bioink containing HDF cells with a density of 2 × 10^6^ cells/mL and cultured for 7 days in an incubator at 37 °C/5%CO_2_. The medium was changed every other day until live/dead assay. On days 1, 3, and 7, the printed lattice constructs were stained with Calcein AM and ethidium homodimer according to the explained protocol and transferred into a confocal dish for taking images.

### Statistics

All measurements were carried out with three replicates and results are expressed as mean ± standard deviation (SD). The Student’s t-test and one-way analysis of variance (ANOVA) with Tukey’s multiple comparison tests were performed in GraphPad Prism software (Version 9.2.0 (332)). The differences with a p-value < 0.05 were considered statistically significant and are indicated with a (*) mark.

## Results

### Biochemical analysis of dAM matrix

The successful removal of cellular and genetic components to an acceptable extent is one of the most critical steps in processing decellularized extracellular matrix (dECM)-derived materials^29^. A credible decellularization process is a delicate matter to retain useful extracellular matrix (ECM) components, such as collagenous proteins and GAGs^30^. In this study, we have chosen a non-ionic detergent (i.e. Triton X-100) and a chelating agent (i.e. EDTA) based on the protocol established by Lee et al.^22^ for decellularization of AM tissue. By quantification of DNA content, it has been found that a significant reduction of cellular components after the decellularization process happens while 65.1 ± 2.5% of collagen and 48.3 ± 2.2% of GAG contents are preserved after the decellularization process (**Figure S1**). These biomolecules are critical elements for the bioactivity of the processed decellularized tissues due to their regulatory function on cell activities^31^. We have reported the histological examination of dAM tissue in our recent publication^12^. The H&E staining of native and decellularized AM exhibited the successful removal of epithelial cells. The Masson’s trichrome and alcian blue stainings showed the preservation of collagen and GAG in the decellularized AM tissue. Lee et al.^22^reported that the current adopted decellularization protocol preserves a significant amount of essential growth factors, including basic fibroblast growth factor (bFGF), vascular endothelial growth factor (VEGF), epidermal growth factor (EGF), transforming growth factor alpha (TGF-α), and beta-1 (TGF-β1), within dAM. The presence of these sets of growth factors within dAM not only encourages cellular activities but also facilitates angiogenesis and tissue regeneration^13^. Overall, the combination of triton X-100 and EDTA seems a proper decellularization process, which does not show harsh effects on the ECM structure and composition, as opposed to other decellularization agents, such as sodium dodecyl sulfate and trypsin^32^.

### Rheological analysis

**Fig. 2:**
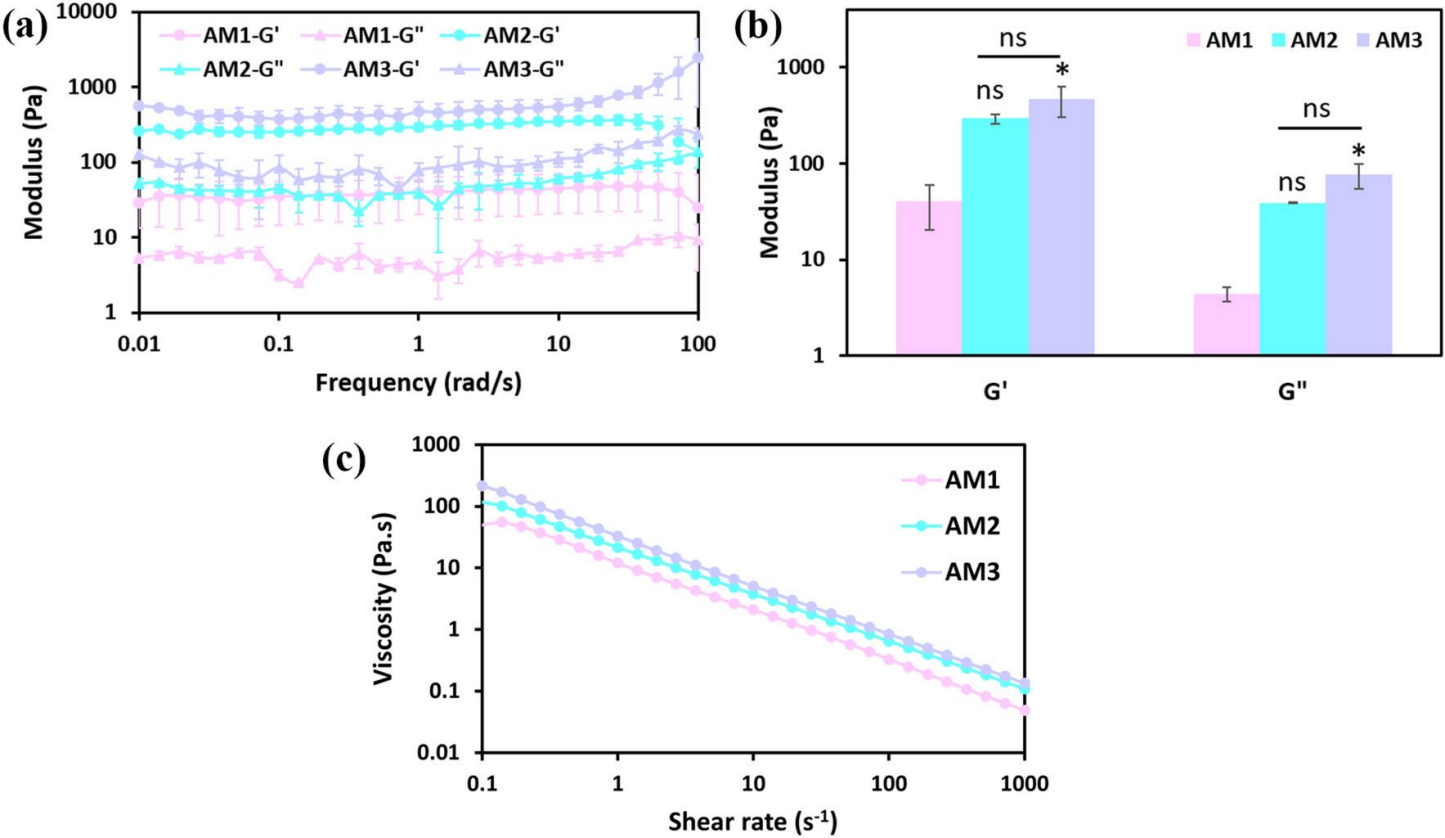
Rheological behavior of hydrogels containing different concentrations of dAM (AM1 represents the hydrogel with 1%w/v dAM content; AM2 represents the hydrogel with 2%w/v dAM content; AM3 represents the hydrogel with 3%w/v dAM content). (**a**) Frequency sweep analysis. (**b**) Effect of dAM concentration on the storage (G") and loss moduli (G") [^*^p < 0.05, ns = no significant difference]. (**c**) Viscosity versus shear rate showing the flow behavior.

The frequency sweep rheological analysis was conducted on thermally crosslinked dAM hydrogels at 37 °C to evaluate the dynamic mechanical moduli. Figure 2a determines the elastic-like nature of dAM hydrogels and their stability under dynamic deformations. The plateau values of storage and loss moduli indicate the remarkable effect of dAM concentration on the mechanical properties. As seen in Fig. 2b, the moduli is enhanced from 40.0 ± 19.6 Pa for AM1 to 471.8 ± 76.7 Pa for AM3. The increased density of collagen fibers within dECM hydrogels contributes to the mechanical properties, as has been observed for other dECM-derived hydrogels^7^. The shear-thinning response of dAM hydrogels is useful for 3D bioprinting (Fig. 2c) because this behavior ensures the survival of cells within bioink^33^. Meanwhile, the more friction forces among polymeric molecules with increasing dAM content enhance the viscosity of dAM pre-gel solutions^8^.

The rheological data were analyzed by three well-known rheological models of Bingham plastic, Casson, and Herschel-Bulkley, and the results are represented in Table 1. The coefficient of determination (R^2^) and fitting results of experimental data to rheological models (**Figure S2**) indicated the suitability of the Herschel-Bulkley to fit the set of experimental results. This model is widely used for explaining the flow behavior of hydrogel solutions because of the consideration of the exponential growth of stress in its formulation^33^. As the consistency coefficient (*k*) of hydrogels depends on their viscosity and internal topography^34^, the enhanced *k* parameter by increasing the concentration of dAM is in good agreement with the observed elevated intrinsic viscosity values of dAM pre-gel solutions (Fig. 2c). Moreover, the critical stress required to initiate the deformation and flow of the hydrogel (i.e., the yield stress, τ_y_) increases with the dAM concentration due to the lower mobility of collagen subunits. It is important to point out that the yield stress contributes to the retention of the injected hydrogel solution placed into a desired defect site by providing stability and immobilization in the absence of a shear force^35^. In this sense, the higher yield stress of hydrogels with high dAM concentration can be favorable for injection into tissue defects^33^and 3D printing^36^ due to better shape fidelity and stability.

**Table 1:** Results of model fitting (Eqs. 1–3) to a set of experimental rheological data.

Hydrogel	Model	k (Pa.s^n^)	n	τ_y_ (Pa)	R^2^
AM1	Bingham plastic	0.043 ± 0.002	-	18.75 ± 0.94	0.646
	Casson	0.118 ± 0.002	-	15.81 ± 0.83	0.824
	Herschel-Bulkley	13.540 ± 0.825	0.19 ± 0.01	0.00 ± 0.00	0.994
AM2	Bingham plastic	0.194 ± 0.010	-	33.45 ± 4.09	0.709
	Casson	0.194 ± 0.009	-	27.34 ± 2.25	0.869
	Herschel-Bulkley	23.060 ± 5.178	0.23 ± 0.01	0.33 ± 0.47	0.995
AM3	Bingham plastic	0.118 ± 0.003	-	46.37 ± 0.73	0.703
	Casson	0.200 ± 0.003	-	38.72 ± 0.56	0.871
	Herschel-Bulkley	28.234 ± 2.425	0.22 ± 0.01	4.56 ± 2.20	0.999

### Printability of bioinks

**Fig. 3:**
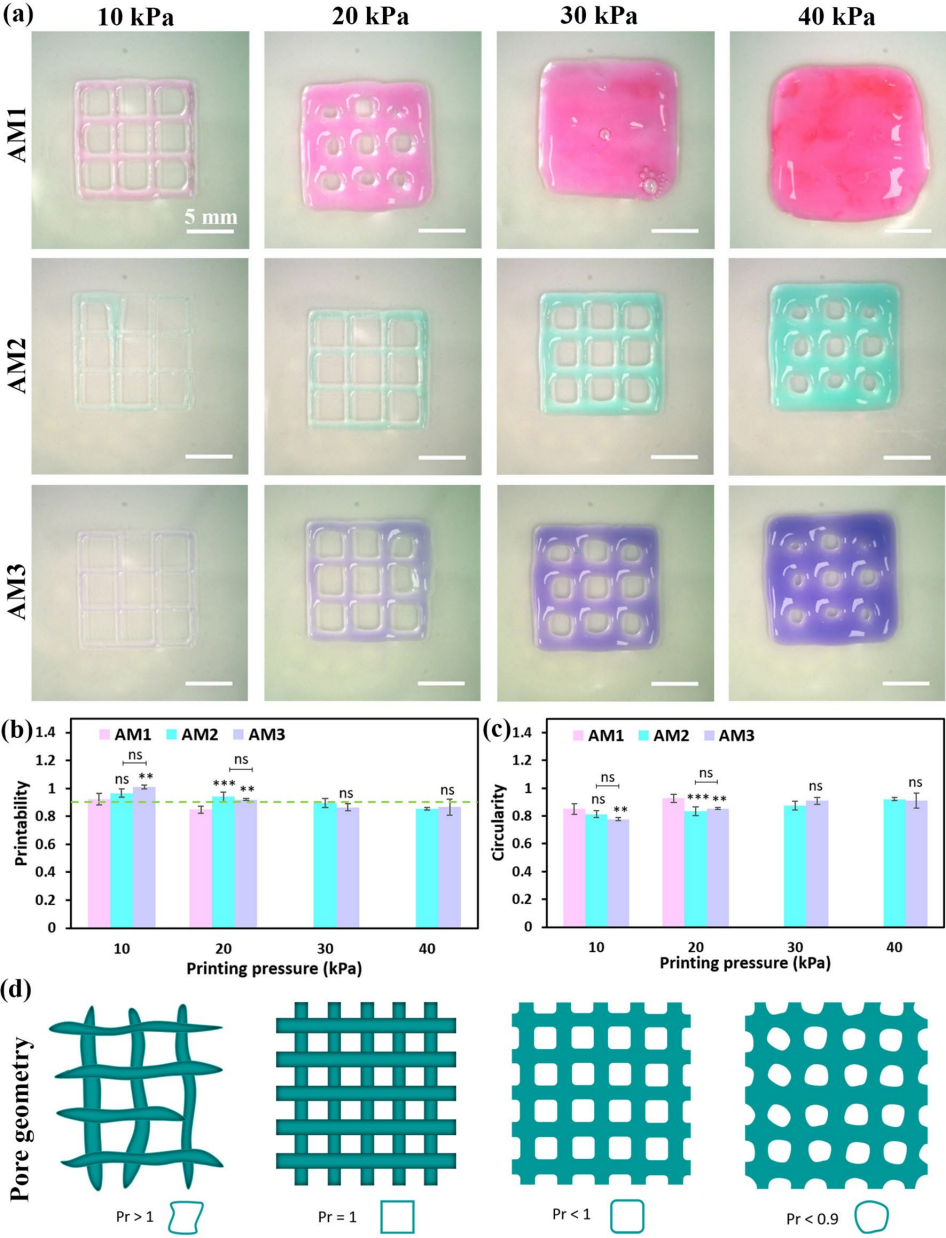
Effect of bioink concentration and printing pressure on 3D printing of lattice structures. (**a**) Representative optical images of printed constructs using dAM bioinks. (**b**) Printability (*Pr*) of lattice structures (The dashed line illustrates the threshold line of *Pr* equal to 0.9, representing reasonable printability beyond that criterion). (**c**) The circularity (*C*) of pores inside the lattice pattern [^**^ *p* < 0.01, ^***^ *p* < 0.001, ns = no significant difference]. (**d**) Representative illustration of 3D printed planar patterns with different printability and their respective pore geometries.

The effect of dAM concentration and printing pressure was examined by evaluating printability (*Pr*) and circularity (*C*) factors of 3D printed lattice structures. Figure 3a shows the optical images of lattice patterns printed at different pressures from 10 to 40 kPa. Notably, the width of printed lines within the lattice pattern for the AM1 bioink is higher than the two other bioinks. This can be related to the lower viscosity of AM1 bioink compared to other counterparts, which allows the deposition of more material under identical printing pressure. This situation intensifies at higher printing pressures, which leads to the closure of designed pore areas inside the lattice pattern. We have found that a lower printing pneumatic pressure and a higher dAM concentration yield a higher printing precision (i.e., strands with a well-defined shape). The printability parameter (*Pr*) is defined as the degree of preserved square-shaped pore area inside the printed lattice patterns^37^. Figure 3b shows that at the constant printing pressure of 10 kPa, the *Pr* value can be ranked as AM3 > AM2 > AM1. Setting *Pr* > 0.9 as the criterion, our results suggest that AM2 and AM3 bioinks offer reasonable printability at 10 or 20 kPa. However, the AM1 bioink can only provide good printability at the lowest tested printing pressure of 10 kPa. As Ouyang et al.^22^ described, a liquid-like state of a bioink with an under-gelation condition leads to the fusion of printed lines causing the designed square pores inside the construct to turn into a circle. Figure 3d presents more details on the relevance of the *Pr* value and the geometry of pores inside printed lattice patterns. Therefore, determining the circularity of pore areas inside the printed pattern can give us an insight into the printability of the developed bioinks. Note that the circularity factor (*C*) for a complete circle is 1.0, and for a square shape is equal to π/4. Figure 3c reveals the effect of dAM concentration and printing pressure on printability by the evaluation of the *C* parameter. We have concluded that the AM3 bioink with printability parameters of *Pr* = 1.01 ± 0.01 and *C* = 0.77 ± 0.01 possesses the best printability at the pressure of 10 kPa.

### Shape fidelity and stacking ability of 3D printed bioinks

**Fig. 4:**
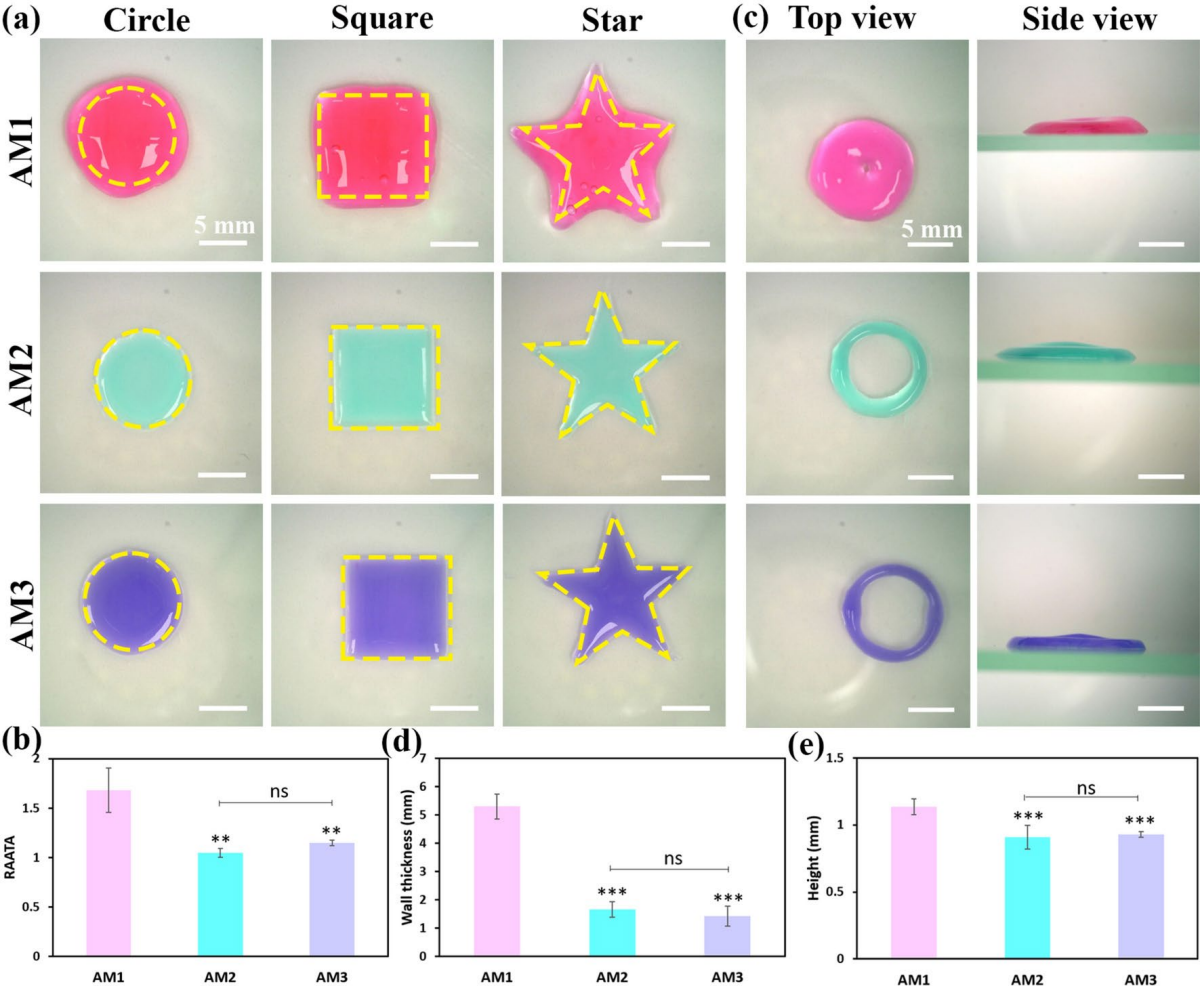
Assessment of shape fidelity and structural integrity of dAM bioinks with different concentrations. (**a**) Representative optical images of the printed constructs (the yellow dash patterns show the borders of the intended original models). (**b**) The ratio of actual area to theoretical area (*RAATA*) for the 3D printed shapes. (**c**) Top-view and side-view images of the printed tubular constructs. (**d**) The wall thickness of printed tubular constructs. (**e**) Height of printed tubular constructs [^**^ *p* < 0.01, ^***^ *p* < 0.001, ns = no significant difference].

To assess the shape fidelity of dAM bioinks, different structures with circle, square, and star shapes were printed. Figure 4a shows that the designed shapes printed by the AM1 bioink are overflowed. The higher dispensing volume of AM1 bioink compared to other counterparts is related to its low viscosity. The AM2 and AM3 bioinks possess better shape fidelity at identical printing conditions (Fig. 4b**)**. A ratio of actual area to theoretical area (*RAATA*) value close to 1.0 represents the similarity of the printed construct to the designed model, which can be attained by using bioinks with a concentration ≥ 2%w/v.

The structural integrity of the developed bioinks was further examined by assessing the stacking ability of bioinks. Tubular constructs with a diameter of 10 mm were printed by deposition of 10 alternative layers on top of each other. Figure 4c exhibits the top- and side-view images of the printed tube constructs. The top-view images determine that the spreading of AM1 bioink leads to the construction of tubes with the highest wall thickness and height with a closed inner space (Fig. 4c-4e). The height of the tubular constructs is shown in Fig. 4e. The height of the constructs ranges between 0.9 to 1.1 mm, showing the spreadable nature of dAM bioinks^38^. This collapsing phenomenon has also been observed for other dECM-derived bioinks^8^ that might have occurred due to the absence of a rapid gelation process to overcome the liquid-like state of the construct immediately after printing. The results suggest that AM1 bioink provides insufficient printability and shape fidelity compared to bioinks with a higher concentration. No statistically significant difference has also been found between AM2 and AM3 bioinks in the concept of printability, shape fidelity, and stacking ability.

## In vitro* studies*

**Fig. 5:**
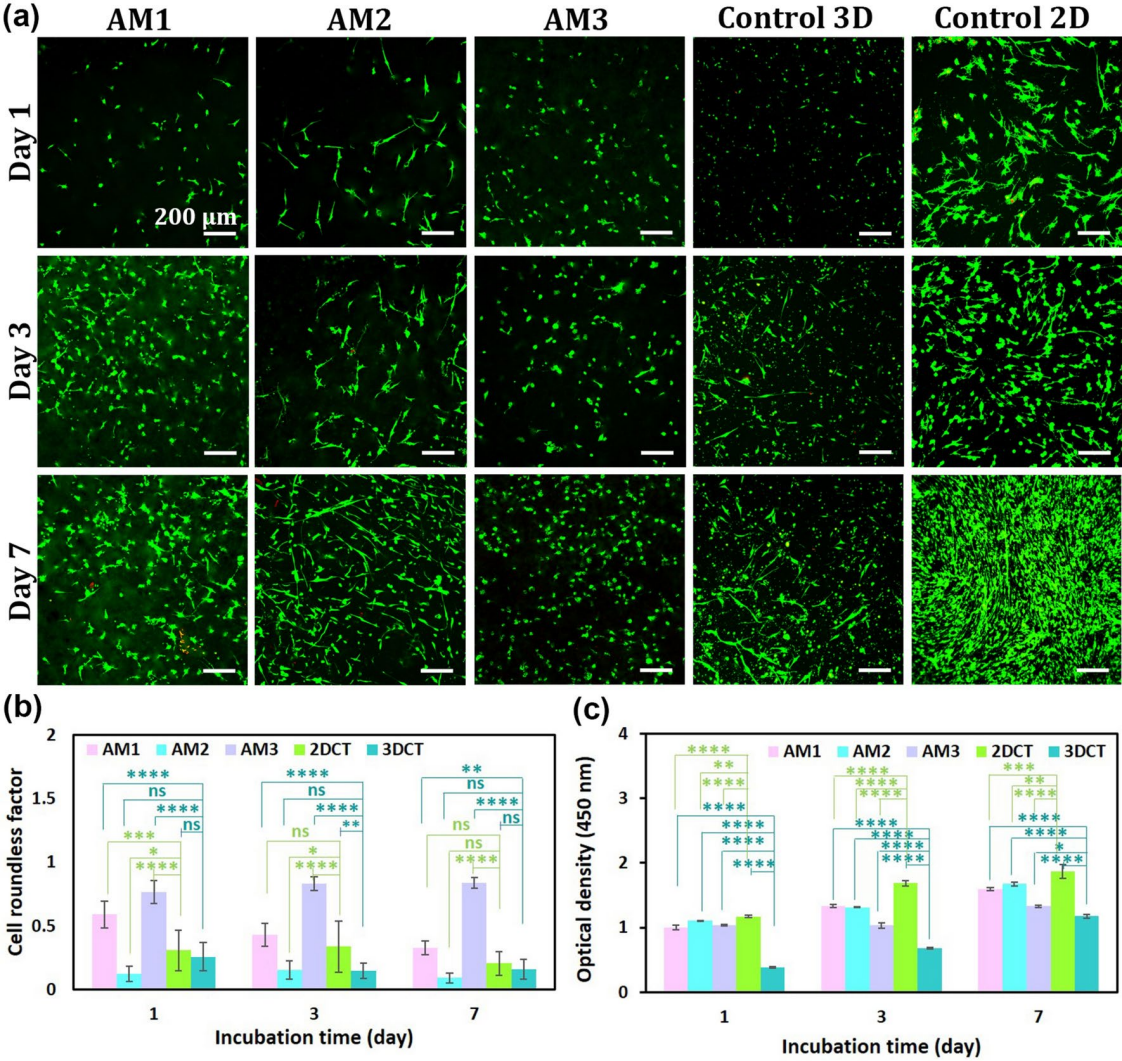
Cytocompatibility assessment of dAM hydrogels with different concentrations. (**a**) Live/dead imaging of HDF cells encapsulated in dAM hydrogels with different concentrations. The HDF cells encapsulated in collagen hydrogel and seeded on the tissue culture plate were imaged as 3D control and 2D control, respectively. (**b**) Quantitative analysis of cell roundness based on live/dead images. (**c**) Cell proliferation of HDF cells encapsulated in dAM hydrogels, 3D control, and 2D control [^*^ *p* < 0.05, ^**^ *p* < 0.01, ^***^ *p* < 0.001, ^****^ *p* < 0.0001, ns = no significant difference].

As represented in Fig. 5a, the live/dead assay was used to evaluate the effect of various dAM concentrations on the viability of the encapsulated HDF cells. The live/dead fluorescence imaging indicates the high viability of HDF cells, as evidenced by a minor proportion of red-stained cells (dead cells) during the 7-day culture period. The cells cultured on a tissue culture plate were used to show the normal morphology of HDF cells in 2D culture. The cells encapsulated in 2%w/v collagen type I were the 3D control group. Notably, the cell morphology was different among testing groups, i.e., the encapsulated HDF cells in AM1, AM2, and AM3 have stretched, elongated spindle-like, and spherical morphologies, respectively (Fig. 5a**).** The HDF cells in the 3D control group represented an elongated morphology, similar to that of AM1 and AM2 hydrogels. These results implied the effectiveness of the dAM hydrogels in providing a safe and bioactive microenvironment to the embedded cells, comparable to standard biocompatible materials, such as collagen. The roundness index of encapsulated cells (Fig. 5b) highlighted the importance of matrix stiffness to affect fibroblast cellular functions^39^. Among all the groups studied, the cells embedded in the AM2 hydrogel exhibited the greatest degree of elongation (indicated by the lowest roundness factor), comparable to that observed in the collagen control group. However, the roundness factor increased by reaching the dAM concentration of 3%w/v (AM3), reflecting the influence of 3D matrix stiffness on restricting the elongation of cells. Studies have determined that a high mechanical modulus (> 1000 Pa) may not support the growth and proliferation of 3D cultured fibroblast cells^40^. Woods et al.^40^ have shown that fibroblast cells encapsulated in microbial transglutaminase crosslinked gelatin hydrogels get a stretched morphology when the elastic modulus is 200–300 Pa and a spherical morphology at an elastic modulus of ~ 1100 Pa.

Figure 5c shows the results of proliferation tests obtained by the CCK-8 method. The data indicate that dAM hydrogels support the proliferative behavior of encapsulated cells, i.e., the proliferation of cells steadily increases over the culture period. The proliferation capacity of encapsulated HDF cells in hydrogels has also been noticed during live/dead assays (Fig. 5a). The comparison of data to the 3D collagen control group also suggests the conducive effect of dAM hydrogels on promoting the proliferation of cells. Yet, the cells embedded in dAM hydrogels seem to be more proliferative compared to merely the collagen control group. This could be attributed to the more complex set of biochemical cues offered by dAM hydrogel, including growth factors, cytokines, GAGs, and other protein constituents as opposed to the singular component of collagen type I^18^. Meanwhile, the proliferation of cells in AM3 hydrogel is significantly lower than that of the other groups. Similar results have been reported for skin-derived bioinks (SdECM) using NIH3T3 mouse fibroblast cells^8^, i.e., a bioink containing 2.5%w/v SdECM exhibits inferior cell viability and proliferation compared to 1.5 and 2%w/v bioinks.

**Fig. 6:**
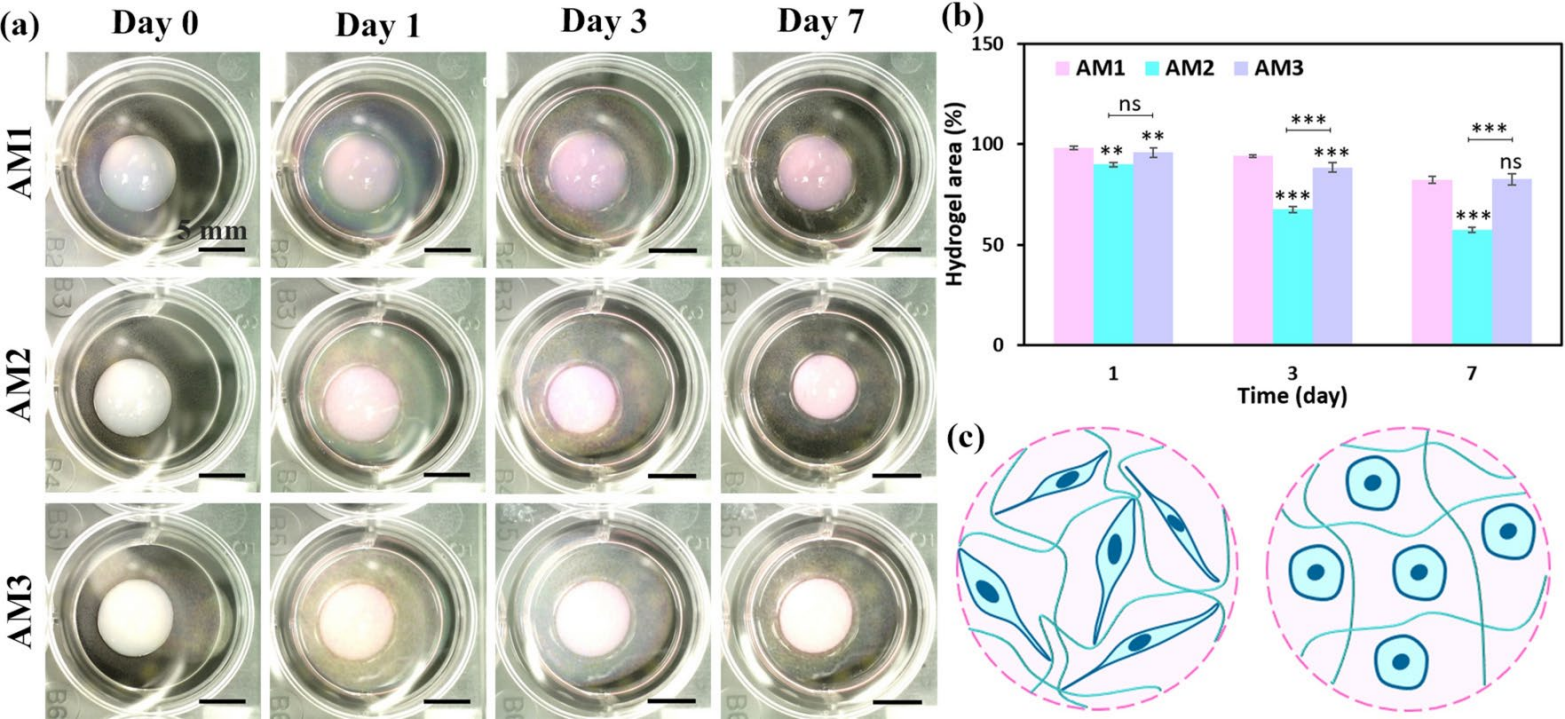
Contraction of HDF-encapsulated dAM hydrogels during 7 days of culture time. (**a**) Macroscopic images of hydrogels at selected time intervals. (**b**) Quantified contraction area determined by image analyzer. (**c**) Schematic representation of the effect of cell morphology on hydrogel contraction and remodeling of ECM network [^**^ *p* < 0.01, ^***^ *p* < 0.001, ns = no significant difference].

The cell-mediated contractility of hydrogels is a feature that affects the remodeling of 3D networks^41^. The cell–matrix contraction is a phenomenon driven by the traction induced by cells, which enables them to reorganize the non-covalent bonds between polymeric chains of the matrix to develop a cellular network by contacting cells^42^. In the current study, we used a gel contraction assay to evaluate the possible effects of matrix stiffness on contractility. The representative images of cell-encapsulated dAM hydrogels and the corresponding changes in hydrogel contraction during a 7-day culture period are shown in Fig. 6a. The quantitative data determine that the contraction gradually increases with the culturing time (Fig. 6b). The highest contraction is attained for the AM2 hydrogel (57.3 ± 1.3% after seven days), which may reflect more active adhesion and remodeling by encapsulated cells^43^. This finding is in agreement with the results of the live-dead assay (Fig. 5a). As schematically illustrated in Fig. 6c, the higher exerted traction forces by elongated HDF cells on collagen fibrils rearrange the dECM network and impose higher contraction over the culture period. It is noteworthy to mention that, despite the superior cellular response of the AM2 hydrogel concerning proliferation and cell morphology, the high contractility might restrict the usage of the hydrogel for some specific TE-based applications. Hence, an additional crosslinking process for the developed dAM hydrogels might be envisioned for better in vitro dimensional retention. Other studies have shown that the contraction of cell-laden dAM hydrogels used for treating cavity-shaped tissue (e.g., dentin pulp) can lead to unfilled spaces over time and mitigate the expected results^44^. Therefore, it is essential to modulate hydrogel systems to minimize contraction while maintaining support for normal elongated cell morphology. Yazdanpanah et al.^45^ have compared thermally crosslinked decellularized corneal-derived hydrogels (COdECM) with the photocrosslinked methacrylated decellularized cornea-derived hydrogels (COdECMMA) alternatives. They have shown that the higher mechanical moduli of the photocrosslinked hydrogels enable the cell-laden constructs to withstand the contracting forces implied by cells with no significant effect on cell viability. Hence, the photocrosslinking of dAM hydrogels seems a practical method to pursue in future studies to combat unintended cell-induced hydrogel contraction.

### Bioprinted constructs

**Fig. 7.**
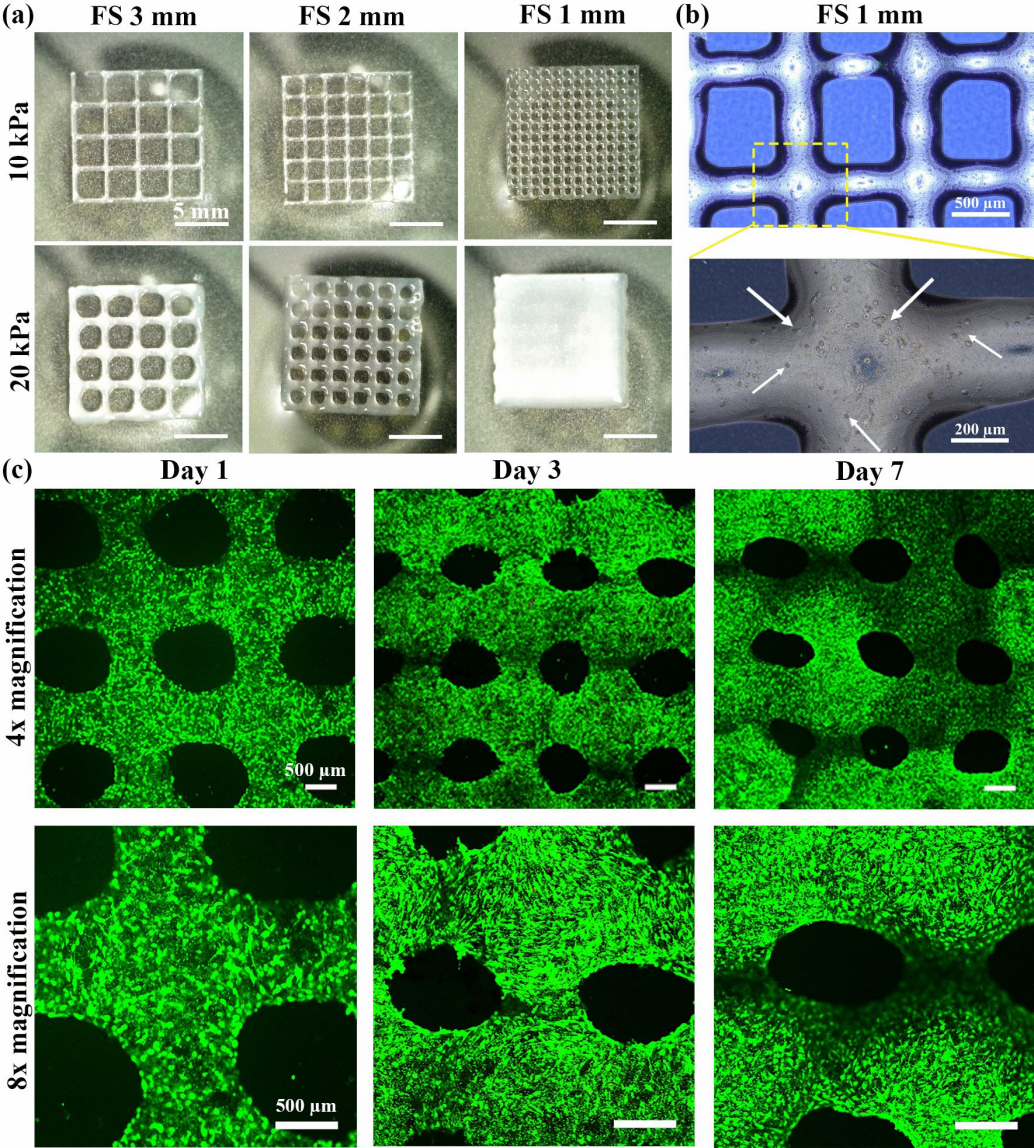
3D bioprinting of lattice construct using AM2 bioink containing HDF cells. (**a**) Evaluation of printing resolution by printing lattice structures with different feature sizes (FS) of 1, 2, and 3 mm using AM2 bioink containing HDF cells with a density of 1 × 10^6^ cells/mL. (**b**) Optical images show the lattice structure of FS1 immediately after 3D bioprinting. The white arrows show the distribution of cells within the construct immediately after bioprinting. (**c**) Results of live/dead assays on bioprinted construct using AM2 bioinks containing HDF cells with a density of 2 × 10^6^ cells/mL after culturing for 1, 3, and 7 days at two different magnifications.

The AM2 bioink was selected for the 3D bioprinting process by the initial screening based on printability and in vitro assays (Figs. 3–5). To evaluate the geometrical precision of bioprinted constructs, lattice patterns with different designed feature sizes (1, 2, and 3 mm) were printed at two pneumatic pressures of 10 and 20 kPa. The images of the printed patterns (Fig. 7a and **Video S1**) determine that lattice patterns with high resolution can be fabricated. Figure 7b demonstrates a representative microscopic image of a printed pattern with a feature size of 1 mm. The images, which are taken immediately after 3D bioprinting, determine that even at such a small feature size, the uniformity and sharp-edged pores are well preserved. It is also notable that cells are distributed evenly within the printed strands. Interestingly, the cells within the 3D bioprinted lattice structure are highly viable after 1, 3, and 7 days of culture (Fig. 7c). The fluorescence images also reveal the stretched morphology of HDF cells. Therefore, the shear forces experienced by cells during the extrusion-based bioprinting process should not impose adverse effects on the viability and cell phenotype. The shear-thinning behavior of the dAM bioink and its bioactivity provide a suitable microenvironment for cell-laden 3D bioprinting.

## Discussion

3D bioprinting is an emerging additive manufacturing technique that allows the deposition of cell-laden bioinks at pre-determined locations to produce the target anatomical architecture^46^. Selecting a bioactive and printable source of cell-encapsulated bioinks in combination with the 3D bioprinting fabrication technique enables the mimicking of both biological and structural features of native tissues. Computed tomography (CT) or magnetic resonance imaging (MRI) of tissue defects further facilitates the biofabrication of patient-specific scaffolds or tissue equivalents through the 3D bioprinting approach^47^. Overall, this biofabrication technique has gained much attention in recent years, thus leading to the development of a wide range of bioinks^48^. Among the various biomaterials suitable as bioinks, dECM hydrogels derived from native tissues have emerged as cell-friendly options for promoting tissue regeneration^49^. In this study, we have chosen dAM as a bioink, owing to its availability and excellent biocompatibility for engineering a wide range of tissues^13^. Nevertheless, there are several critical considerations, such as rheological properties, printability, and cell compatibility, for developing a bioink formulation^28^. A proper bioink should provide sufficient rheological properties enabling extrudability, post-printing shape fidelity, and structural integrity. We have studied the printability of developed bioinks by 3D printing different lattice patterns for planar structures and evaluated the shape fidelity and stacking ability for multilayer constructs. It has been found that under the same printing conditions, 1%w/v dAM bioink exhibits an excessive dispensing volume due to its low viscosity. However, at higher concentrations (≥ 2%w/v) a superior printability is attained, as evidenced by sound strand morphology and distinguishable lattice patterns with sharp-edged square pores. The calculated shear index (n) value less than 1.0 for all examined dAM hydrogels determines a shear-thinning characteristic that benefits cell survival during 3D bioprinting (Table 1**, **Fig. 7c). The viscosity of a bioink is also an important determinant for predicting printability. Our results have revealed that AM1 bioink with the lowest viscosity value exhibits undesirable printability and shape fidelity (Figs. 3 and 4). On the other hand, as a result of enhanced viscosity, better geometric fidelity of planar patterns can be obtained for both AM2 and AM3 hydrogels as opposed to the AM1 counterpart. The yield stress of a bioink is another prerequisite factor affecting stacking ability. The rheological analysis has shown that increasing the dAM concentration from 1 to 3% enhances the average yield stress from 0 to 4.5 Pa (Table 1). As shown in Fig. 4c**,**the dAM bioinks face a limitation in stacking ability in the Z-direction due to their intrinsic spreading characteristic and a lack of a rapid gelation process. Based on previous studies^33^, an excellent stacking ability of a bioink requires a yield stress higher than 100 Pa. Therefore, a high stacking ability of the dAM bioinks is not expected^8^. Meanwhile, studies have shown that the incorporation of sodium alginate, as a structurally supportive component, and Laponite nanoplatelet, as a rheology modifier, facilitates 3D printing of self-standing tubular constructs^23^. The main shortcoming of sodium alginate is the lack of cell-binding motifs that diminish the bioactivity of the final TECs, whilst aggregation and nonhomogeneous distribution of Laponite particles in the hydrogel matrix is challenging. Our findings propose the necessity of considering emerging strategies, such as rapid crosslinking of dECM bioinks through photochemistry^50^, for bioprinting of complex-shaped 3D structures.

Adjusting the rheological properties of a bioink to enhance printability and shape fidelity is a delicate balance, as optimizing these features can often compromise cytocompatibility^51^. To investigate the effect of the mechanical modulus of thermally crosslinked dAM hydrogels in cell viability, we have employed live/dead assays during 7 days of culture. It is noteworthy that fibroblast cells effectively regulate wound healing by contributing to ECM secretion and the remodeling process^52^. The viability of HDF cells seems to be independent of dAM concentration and all hydrogels provide a suitable microenvironment for cells to survive during the 7-day culture period (Fig. 5a). Nevertheless, the tuning of hydrogel’s mechanical moduli may lead to different cell morphologies. As shown in Fig. 5, increasing the concentration of dAM from 1%w/v to 2%w/v indicates more elongated fibroblast cells. The elongated morphology and viability of HDFs are similar to that in collagen, which is a widely used biocompatible hydrogel. At the higher concentration (3%w/v), however, an almost round cell morphology is adopted after culturing for seven days. We propose that the lower capability of cells to remodel in the AM3 hydrogel is attributed to the higher stiffness of the matrix^40^. It is known that the regulation of cell spreading in 3D culture due to matrix stiffness is highly affected by the ability of cells to remodel the surrounding microenvironment^53^. As reported by Caliari et al.^54^, changing the matrix stiffness significantly affects the spreading of encapsulated mesenchymal stem cells (MSC) in hyaluronic acid hydrogels with varied stiffness by activating the YAP/TAZ (Yes-associated protein/ Transcriptional coactivator with PDZ binding motif) mechanosensitive signaling pathway. The lower stiffness of hydrogels also facilitates cell mobility and spreading^55^. Our results have also revealed that HDF cells encapsulated in all dAM hydrogels remain viable and proliferative during the seven-day culture period (Fig. 5c). Meanwhile, the cells are less proliferative in the AM3 hydrogel due to the high elastic modulus. Cell proliferation is known to be affected by cell spreading^40^and cell–cell contacts^56^. The results of live/dead staining show fewer cell–cell contacts in the AM3 hydrogel (Fig. 5a); hence, the cells are less proliferative. Overall, the results determine that the AM2 hydrogel provides a suitable biomimetic microenvironment for cell-laden bioprinting because HDF cells preserve their viability and adopt stretched morphology within the bioprinted constructs. The high cell viability can partly be related to its low shear stress value that hampers the risk of high cell death during extrusion-based bioprinting. The preliminary biological assays performed in this study support the bioactivity and cell compatibility of dAM bioink for TE applications. Yet, further sophisticated biological assays, including immunostainings and histological examinations, are warranted to establish the adaptability of dAM for specific biomedical applications and pave its way to translational medicine.

Although the contraction of cell-laden hydrogels represents the remodeling activity of the embedded cells, it may confer with the targeted in vivo regenerative applications. The cell-mediated contraction of hydrogels is a factor that impacts the size of graft tissue-engineered substitutes leading to separation from host tissue and subsequently influencing the reconstruction of neotissues^57^. In agreement with our results (Fig. 6**), **cell-induced contraction has been observed in collagen-based and dECM-derived hydrogels. Although chemical crosslinking of hydrogels can mitigate the tension arising from cell–matrix interactions, the utilized agents are highly susceptible to causing cell cytotoxicity and escalating immune responses^58^. Therefore, enhancing the mechanical properties by increasing the concentration of collagen-based hydrogels has been proposed as an alternative approach to dealing with cell-induced contraction phenomena^59^. We have used the concentration-modulated approach to HDF-embedded dAM hydrogels to investigate the cell–matrix interactions. The results propose that increasing the concentration of physically crosslinked dAM hydrogels provides better mechanical stability. However, the higher stiffness of hydrogels may interfere with the uniform re-assembling of collagenous subunits and impede the morphological and biological functionality of the encapsulated cells (e.g. comparison of AM3 with AM2 hydrogel). Rothrauff et al.^21^have shown that photocrosslinking of methacrylate-functionalized decellularized cartilage (CdECMMA) or tendon-derived hydrogels (TdECMMA) can enhance their resistance to cell-induced contraction, as opposed to non-methacrylated counterparts. Thereupon, it is recommended to consider photocrosslinking of dAM bioinks to gain more control over cell-mediated contraction. This approach is also anticipated to enhance the stacking ability of dAM bioinks by providing structural stability to printed constructs through light exposure on consecutive deposited layers. On the other hand, chemical modification of dECM biomaterials should be pursued cautiously since these alterations may diminish the bioactivity of the dECM matrix and interfere with the target tissue-specific functionality^21^.

It is worth mentioning that to achieve the highest degree of mimicry, it is highly suggested utilizing the same source of dECM as the injured tissue to ensure better integration of TEC with the surrounding host tissue^60^. Yet, sometimes it is also a rational choice to select another available tissue source with substantial compatibility with the ECM composition of target tissue. For example, the methacrylated decellularized Wharton’s jelly (WdECMMA) was introduced as a reliable source to regenerate the cartilage rings of the trachea^49^. The selection of Wharton’s jelly was due to the resemblance of hyaluronic acid, collagen, GAG, and growth factors, such as insulin-like growth factor-1 (IGF-1) and transforming growth factor beta (TGF-β) to that of cartilage tissue. In line with this study, the tissues derived from the placenta, such as the Wharton’s jelly and amniotic membrane hold a particular position due to their abundance, ease of access, and biocompatibility^61,62^. The amniotic membrane is a unique source of tissue that has been shown favorable biomedical and regenerative results for treating a wide range of tissue injuries, including skin, cornea, heart, liver, vasculature, endometrium, cartilage, and dental pulp^13^. Particularly, the amniotic membrane possesses a biochemical composition similar to the skin tissue, making it a promising candidate for skin regeneration and wound healing applications^63^. While the tissue-specificity of skin dECM for wound regeneration may surpass that of dAM tissue, dAM still offers distinct advantages, including low immunogenicity, high cell compatibility, and broad availability, setting it apart from other alternatives. Specifically, dAM hydrogels show promising therapeutic outcomes in treating skin wounds, thanks to their bioactivity, ability to regulate inflammatory responses, and promotion of angiogenesis^14,64^. Studies have demonstrated promising outcomes as a carrier of various stem cells by supporting their viability, proliferation, and stemness^29^. These cell-encapsulating vehicles provide promising biological functionality as compared with the gold standard treatments, such as fibrin gel and collagen hydrogel while exhibiting even less in vivo immunogenicity^18^. Nevertheless, the potential of dAM bioinks without having any auxiliary compounds has not been evaluated in the literature. The current study provides insight into the printability of dAM bioinks and skin-relevant cell type responses to the microenvironment provided by the dAM matrix. Although our results support the concentration-dependent printability of dAM bioinks, future studies are required to support their biologically relevant responses. In this context, dAM bioinks containing keratinocyte and fibroblast cells can be envisioned for bioprinting of epidermal-dermal substitutes. These types of tissue equivalents can be used for wound repair and developing tissue modeling platforms for screening drugs or cosmetics. It has been reported that the resolution of bioprinted constructs affects the proliferation of skin cells^65^. Our study has shown that a dAM bioink with an adjusted concentration provides high printing resolutions with small feature sizes in planar constructs (Fig. 7a) that may benefit the biofabrication of dermal skin mimetics with proliferative cells. It is noteworthy that dAM has been utilized as a gold standard clinical procedure in treating different corneal injuries and defects, owing to its biocompatibility, immune privilege, inherent regenerative growth factors, cytokines, and quite low opacity^66^. Nevertheless, its weak mechanical stability, difficult handling, and high biodegradability rate restrict its grafting outcome^67^. To address this challenge, 3D bioprinted corneal equivalents using dAM bioinks can be used to treat patient-specific corneal defects, similar to 3D printed decellularized cornea-derived bioinks^68^. Biomimetic inks, such as corneal-specific cell types encapsulated in dAM hydrogels, also can be employed to fabricate multilayered corneal structures replicating the anatomical architecture of normal corneal tissue. Furthermore, the application of 3D bioprinted dAM bioink for imitating the intertwined collagen arrangement in the anterior part of the cornea as well as the aligned organization of fibrous components in the middle and posterior parts of the cornea may accomplish more structurally relevant features similar to native cornea^69^. Henry et al.^15^ have introduced the application of an injectable dAM hydrogel for treating myocardium after myocardial infarction (MI) by promoting angiogenesis and exhibiting antifibrotic characteristics. Thus, the dAM bioink seems to be a suitable option for 3D printing of patterned cardiac patches for repairing MI-induced tissue defects. However, the low mechanical durability of printed patches requires further improvement or accompanying with a supporting polymeric framework to endure the constant contractile motions of the heart. Overall, we propose that a dAM bioink with proper concentration can be applied for 3D bioprinting of plane tissue equivalents, such as skin or ocular tissue, by mimicking both the geometrical and biochemical features of the respective native tissues. However, the thermally crosslinked dAM bioink may not provide sufficient characteristics for the bioprinting of geometrically sophisticated tissues/organs due to the time-consuming gelation process. Therefore, modification of dAM bioink to provide higher rheological and mechanical properties to withstand the weight of alternative layers bioprinted on top of each other while not harming cellular functions can be pursued in future research.

## Conclusions

This study investigated the rheological properties and printability of enzymatically digested dAM bioinks as a function of dAM concentration. The dynamic mechanical moduli of the bioinks increased with concentrations ranging from 1% w/v to 3% w/v. The higher viscosity observed at concentrations of 2% w/v and above facilitated the proper shape fidelity of printed flat patterns. However, similar to other dECM bioinks, the printing of cell-laden constructs with alternating layers proved challenging due to relatively low yield stress. In vitro studies indicated that the cells were viable and proliferative over seven days of culture in all dAM concentrations. The bioink containing 2%w/v dAM exhibited the highest cell proliferation rate with spindle-like cell morphology. The 3D bioprinting of HDF cell-laden constructs determined that delicate structures with high lateral resolution and cell compatibility could successfully be bioprinted for soft tissue engineering. More studies are underway to elaborate on specific biological applications of the developed dAM bioinks.

### Supporting information

The supplementary information presents the biochemical assays of AM tissue after decellularization and the model fitting of the rheological data.

## Supplementary Information

Supplementary Information.

## Data Availability

The raw/processed data required to reproduce these findings are available for sharing by Golara Kafili (g_kafili@yahoo.com) upon a reasonable request.
