# Supplementary material for: The impact of mechanical tuning on the printability of decellularized amniotic membrane bioinks for cell-laden bioprinting of soft tissue constructs
Source: Sci Rep. 2024 Nov 29;14:29697. doi: 10.1038/s41598-024-80973-3 (PMC11606975; doi:10.1038/s41598-024-80973-3)
Supplement: Supplementary file 1 — Supplementary Information. [file 41598_2024_80973_MOESM1_ESM.docx]

Supplementary data

**The impact of mechanical tuning on the printability of decellularized amniotic membrane bioinks for cell-laden bioprinting of soft tissue constructs**

Golara Kafili^1^, Elnaz Tamjid^2,3^, Abdolreza Simchi^1, 4, 5^*†

^1^ Center for Nanoscience and Nanotechnology, Institute for Convergence Science & Technology, Sharif University of Technology, P.O. Box 14588-89694, Tehran, Iran.

^2^ Department of Nanobiotechnology, Faculty of Biological Sciences, Tarbiat Modares University, P.O. Box 14115-175, Tehran, Iran.

^3^ Advanced Ceramics, University of Bremen, 28359 Bremen, Germany

^4^ Department of Materials Science and Engineering, Sharif University of Technology, P.O. Box 11365-11155, Tehran, Iran.

^5^ Center for BioScience and Technology, Institute for Convergence Science & Technology, Sharif University of Technology, P.O. Box 14588-89694, Tehran, Iran.

Email address: [g_kafili@yahoo.com](mailto:g_kafili@yahoo.com) (G. Kafili), [tamjid@modares.ac.ir](mailto:tamjid@modares.ac.ir) (E. Tamjid)

^*^ Corresponding author: Abdolreza Simchi

Department of Materials Science and Engineering, Sharif University of Technology, Azadi Avenue, P.O. Box 11365-8639, Tehran, Iran. Tel: +98-21-66165261; Fax: +98-21-66005717. Email: [simchi@sharif.edu](mailto:simchi@sharif.edu)

† Current affiliation: Fraunhofer Institute for Manufacturing Technology and Advanced Materials (IFAM), 28359 Bremen, Germany; Email: [abdolreza.simchi@ifam.fraunhofer.de](mailto:abdolreza.simchi@ifam.fraunhofer.de)


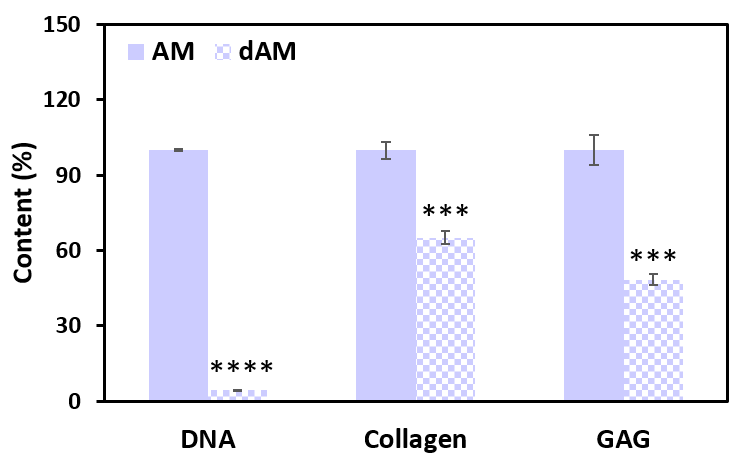


**Figure S1.** Biochemical analysis of the dAM matrix. Effect of decellularization on the content of DNA, collagen, and GAG in the AM tissue. The *p*-value was determined by Student’s t-test [^***^ *p* ˂0.001, ^****^ *p* ˂0.0001, ns = no significant difference].


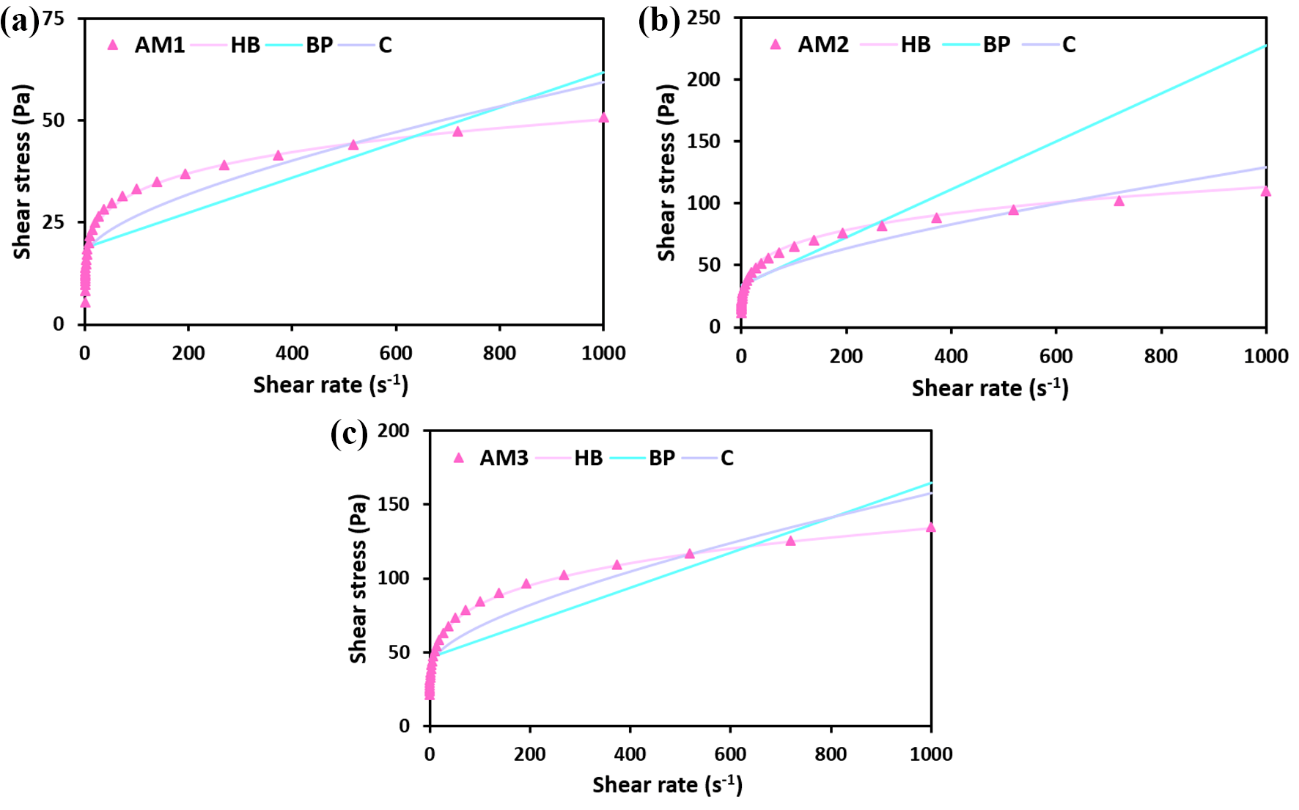


**Figure S2.** Fitting experimental rheological data of hydrogels with different concentrations: (a) 1 %w/v dAM; (b) 2 %w/v dAM; (c) 3 %w/v dAM. The utilized rheological models were Herschel-Bulkley (HB), Bingham plastic (BP), and Casson (C).
